# A New First-Order Integer-Valued Autoregressive Model with Bell Innovations

**DOI:** 10.3390/e23060713

**Published:** 2021-06-04

**Authors:** Jie Huang, Fukang Zhu

**Affiliations:** School of Mathematics, Jilin University, 2699 Qianjin Street, Changchun 130012, China; jiehuang19@mails.jlu.edu.cn

**Keywords:** Bell distribution, count time series, estimation, INAR, overdispersion

## Abstract

A Poisson distribution is commonly used as the innovation distribution for integer-valued autoregressive models, but its mean is equal to its variance, which limits flexibility, so a flexible, one-parameter, infinitely divisible Bell distribution may be a good alternative. In addition, for a parameter with a small value, the Bell distribution approaches the Poisson distribution. In this paper, we introduce a new first-order, non-negative, integer-valued autoregressive model with Bell innovations based on the binomial thinning operator. Compared with other models, the new model is not only simple but also particularly suitable for time series of counts exhibiting overdispersion. Some properties of the model are established here, such as the mean, variance, joint distribution functions, and multi-step-ahead conditional measures. Conditional least squares, Yule–Walker, and conditional maximum likelihood are used for estimating the parameters. Some simulation results are presented to access these estimates’ performances. Real data examples are provided.

## 1. Introduction

In recent years, studying count time series has attracted a lot of attention in different fields, such as finance, medical science, and insurance. There are many models for count data that have been proposed by scholars. The most famous model was first introduced by McKenzie (1985) [1] and Al-Osh and Alzaid (1987) [2] based on the binomial thinning ∘ operator (Steutel and van Harn 1979 [3]) called the first-order integer-valued autoregressive (INAR(1)) process. Given a non-negative integer-valued random variable (r.v.) *X* and a constant α∈(0,1), the binomial thinning operator ∘ is defined as $\alpha \circ X=\sum_{i=1}^{X}\xi_{i}$, where the counting series $\xi_{i}$ is a sequence of independent identically distributed (i.i.d.) Bernoulli r.v.s with $P\left(\xi_{i}=1\right)=1-P\left(\xi_{i}=0\right)=\alpha$. Then, the form of the INAR(1) model is

(1) $$X_{t}=\alpha \circ X_{t-1}+\epsilon_{t},\;\;t=0,1,2,\ldots ,$$

where $\epsilon_{t}$ is a sequence of i.i.d. discrete r.v.s, with the mean $\mu_{\epsilon }$ and finite variance $\sigma_{\epsilon }^{2}$. $\epsilon_{t}$ is independent of $\xi_{i}$ and $X_{t-s}$ for s≥1. According to Alzaid and Al-Osh (1988) [4], we know that the mean and variance of the INAR(1) model are

$$\mu :=\mu_{X}=\frac{\mu_{\epsilon }}{1-\alpha }\;\mathrm{and}\;\sigma^{2}:=\sigma_{X}^{2}=\frac{\sigma_{\epsilon }^{2}+\alpha \mu_{\epsilon }}{1-\alpha^{2}},\;\mathrm{respectively}.$$

For innovation $\epsilon_{t}$, the Poisson distribution is often assumed as the distribution of $\epsilon_{t}$ in the INAR(1) model. A natural characteristic of the Poisson distribution is equidispersion; i.e., its mean and variance are equal to each other. In practice, however, many data examples are overdispersed (variance is greater than mean) relative to the Poisson distribution. For this reason, the INAR(1) model with Poisson innovations is not always suitable for modeling integer-valued time series. Therefore, several models which describe the overdispersion phenomena have been discussed in the statistical literature.

One common approach is to change the thinning operation in the INAR(1) model. Weiß (2018) [5] summarized several alternative thinning operators, such as random coefficient thinning, iterated thinning and quasi-binomial thinning. Ristić et al. (2009) [6] proposed the negative binomial thinning operator and defined the corresponding INAR(1) process with geometric marginal distributions. Liu and Zhu (2021) [7] generalized the binomial thinning operator to the extended binomial one.

Changing the distribution of innovations is also used to modify the INAR(1) model. Jung et al. (2005) [8] indicated that the INAR(1) model with negative binomial innovations (NB-INAR(1)) is appropriate for generating overdispersion. Jazi et al. (2012) [9] defined a zero-inflated Poisson ZIP(ρ, λ) for innovations (ZIP-INAR(1)), because a frequent occurrence in overdispersion is that the incidence of zero counts is greater than expected from the Poisson distribution. Jazi et al. (2012) [10] proposed a modification of the INAR(1) model with geometric innovations (G-INAR(1)) for modeling overdispersed count data. Schweer and Weiß (2014) [11] investigated the compound Poisson INAR(1) (CP-INAR(1)) model, which is suitable for fitting datasets with overdispersion. According to Schweer and Weiß (2014) [11], we can also know that the negative binomial distribution and the geometric distribution both belong to the compound Poisson distribution. Livio et al. (2018) [12] presented the INAR(1) model with the Poisson–Lindley innovations, i.e., PL-INAR(1). Bourguignon et al. (2019) [13] introduced the INAR(1) model with the double Poisson (DP-INAR(1)) and generalized Poisson innovations (GP-INAR(1)). Qi et al. (2019) [14] considered zero-and-one inflated INAR(1)-type models, and Cunha et al. (2021) [15] introduced an INAR(1) model with Borel innovations to model zero truncated count time series.

This paper applies the second approach to dealing with overdispersion. Although several models have been proposed in recent years, most of the considered distributions are based on some generalizations of the Poisson distribution and have more than one parameter, such as the zero-inflated Poisson, compound Poisson, double Poisson, and generalized Poisson distributions. Here we use a relatively simple distribution introduced by Castellares et al. (2018) [16] for the innovations, i.e., the Bell distribution. It has the advantages of having only one parameter, belonging to the exponential family, having a simple probability mass function, and having infinite divisibility. Infinite divisibility is significant for constructing the binomial thinning INAR(1) model. Further, the Bell distribution is suitable for modeling some overdispersed count data. Therefore, we introduce a new INAR(1) model with Bell innovations (BL-INAR(1)), which can account for overdispersion in an INAR(1) framework.

In order to observe whether the BL-INAR(1) model has advantages, we compare it with the INAR(1) model with Poisson innovations (P-INAR(1)), G-INAR(1), PL-INAR(1), NB-INAR(1), ZIP-INAR(1), DP-INAR(1), and GP-INAR(1) models. Different information criteria, such as Akaike’s information criterion (AIC) [17], the Bayesian information criterion (BIC) [18], the consistent Akaike information criterion (CAIC) [19], and the Hannan–Quinn information criterion (HQIC) [20], are used to compare the above eight models. By comparing the results of different information criteria, it can be seen that the BL-INAR(1) model is competitive when modeling the overdispersed integer-valued time series data, which shows that the proposed BL-INAR(1) model is meaningful; see Section 5 for more details.

We organize the remaining parts of this paper as follows. In Section 2, we briefly review the Bell distribution, including its definition and some properties. Then we propose the BL-INAR(1) model, and its basic properties are constructed; conditional mean and variance are obtained. Section 3 discusses estimates of the model parameters by using the conditional least squares (CLS), Yule–Walker (YW), and conditional maximum likelihood (CML) methods. In Section 4, a numerical simulation of the estimates is presented with some discussions. In Section 5, we compare the proposed model with the other seven INAR(1)-type models when fitting two real data examples, which show the superior performances of the proposed model. The paper concludes in Section 6.

## 2. The BL-INAR(1) Model

In this section, we present a brief review of the Bell distribution (Castellares et al., 2018 [16]). Its definition and some properties are presented. Later we introduce the BL-INAR(1) model and derive some basic properties of it.

### 2.1. The Bell Distribution

At first, we introduce the Bell numbers. Bell (1934) [21] has provided the following expansion:

$$\exp\left(e^{x}-1\right)=\sum_{n=0}^{\infty }\frac{B_{n}}{n!}x^{n},\;x\in R,$$

where $B_{n}$ is the Bell number defined by

(2) $$B_{n}=\frac{1}{e}\sum_{k=0}^{\infty }\frac{k^{n}}{k!}.$$

The Bell number $B_{n}$ is the *n*-th moment of the Poisson distribution with parameter equal to 1. Some Bell numbers are listed as follows. $B_{0}=B_{1}=1,B_{2}=2,B_{3}=5$, $B_{4}=15$, $B_{5}=52,B_{6}=203,B_{7}=877,B_{8}=4140,B_{9}=$ 21,147, $B_{10}=$ 115,975, $B_{11}=$ 678,570, $B_{12}=$ 4,213,597 and $B_{13}=$27,644,437.

For the convenience of the reader, we introduce the following definition and properties of the Bell distribution described in Castellares et al. (2018) [16]:

**Definition** **1.** *A discrete r.v. Z taking values in $N_{0}=\left\{0,1,2,\ldots \right\}$ has a Bell distribution with parameter θ>0, denoted as Z∼Bell(θ), if its probability mass function is given by*

(3) $$\mathrm{Pr}\left(Z=z\right)=\frac{\theta^{z}e^{-e^{\theta }+1}B_{z}}{z!},\;z\in N_{0},$$

*where $B_{z}$ is the Bell number in (2).*

We can see that the Bell distribution has only one parameter, and it belongs to the one-parameter exponential family of distributions. If Z∼Bell(θ), the probability generating function is

$$G_{Z}\left(s\right)=E\left(s^{Z}\right)=\exp\left(e^{s\theta }-e^{\theta }\right),\;\left|s\right|<1.$$

The mean and variance of *Z* are

(4) $$E\left(Z\right)=\theta e^{\theta }\;\mathrm{and}\;\mathrm{Var}\left(Z\right)=\theta \left(1+\theta \right)e^{\theta },\;\mathrm{respectively}.$$

Note that Var(Z)/E(Z)=1+θ>1; hence, the Bell distribution is overdispersed, which means the Bell distribution may be suitable for count data with overdispersion in certain situations.

There are some other interesting properties of the Bell distribution, including the following: (i) the Poisson distribution is not nested in the Bell family, but for small values of the parameter, the Bell distribution approaches the Poisson distribution; (ii) it is identifiable, strongly unimodal and infinitely divisible; (iii) a r.v. Z∼Bell(θ) has the same distribution as $Y_{1}+Y_{2}+\cdots +Y_{N}$, where $Y_{n}$ has zero-truncated Poisson distribution with parameter θ, and $N∼\mathrm{Poisson}(e^{\theta }-1)$. See Castellares et al. (2018) [16] for more properties.

Additionally, there are some papers based on the Bell distribution, and the following are a few related references: Batsidis et al. (2020) [22] proposed and studied a goodness-of-fit test for the Bell distribution, which is consistent against fixed alternatives; Castellares et al. (2020) [23] presented a new two-parameter Bell–Touchard discrete distribution; Lemonte et al. (2020) [24] introduced a zero-inflated Bell regression model for count data; Muhammad et al. (2021) [25] proposed a Bell ridge regression as a solution to the multicollinearity problems.

### 2.2. Definition and Properties of the BL-INAR(1) Process

In this section, we give the definition of the BL-INAR(1) process, and its basic statistical properties are derived.

**Definition** **2.** *Let ${\left\{X_{t}\right\}}_{t\in N_{0}}$ be an INAR(1) process according to (1). It refers to a BL-INAR(1) model if the innovations ${\left\{\epsilon_{t}\right\}}_{t\in N_{0}}$ are a sequence of i.i.d. Bell(θ) r.v.s given by (3); i.e.,*

(5) $$\left\{\begin{array}{c} X_{t}=\alpha \circ X_{t-1}+\epsilon_{t},\;\;\;t\geq 1,\\ \epsilon_{t}∼\mathrm{Bell}\left(\theta \right), \end{array}\right.$$

*where 0<α<1 and θ>0, and $\epsilon_{t}$ is independent of $\xi_{i}$ and $X_{t-1}$ for t≥1.*

According to Equation (4), we know the mean and variance of $\epsilon_{t}$ are finite; therefore, the process of ${\left\{X_{t}\right\}}_{t\in N_{0}}$ in (5) is an ergodic stationary Markov chain (Du and Li, (1991) [26]) with transition probabilities

$$\begin{array}{cc} P_{ij} & =P\left(X_{t}=i|X_{t-1}=j\right)=P\left(\alpha \circ X_{t-1}+\epsilon_{t}=i|X_{t-1}=j\right)\\ \; & =\sum_{m=0}^{\min(i,j)}P\left(\alpha \circ X_{t-1}=m|X_{t-1}=j\right)P\left(\epsilon_{t}=i-m\right)\\ \; & =\sum_{m=0}^{\min(i,j)}\left(\begin{array}{c} j\\ m \end{array}\right)\alpha^{m}{\left(1-\alpha \right)}^{j-m}\frac{\theta^{i-m}e^{-e^{\theta }+1}B_{i-m}}{(i-m)!},\;i,j=0,1\ldots \end{array}$$

Further, we can obtain the joint probability function as follows:

(6) $$\begin{array}{cc} f\left(i_{1},i_{2}\ldots i_{T}\right) & =P\left(X_{1}=i_{1},X_{2}=i_{2},\ldots ,X_{T}=i_{T}\right)\\ \; & =P\left(X_{1}=i_{1}\right)P\left(X_{2}=i_{2}|X_{1}=i_{1}\right)\ldots P\left(X_{T}=i_{T}|X_{T-1}=i_{T-1}\right)\\ \; & =P\left(X_{1}=i_{1}\right)\prod_{k=1}^{T-1}\left[\sum_{m=0}^{\min\left(i_{k},i_{k+1}\right)}\left(\begin{array}{c} i_{k}\\ m \end{array}\right)\alpha^{m}{\left(1-\alpha \right)}^{i_{k}-m}P\left(\epsilon_{k+1}=i_{k+1}-m\right)\right]. \end{array}$$

The conditional mean, conditional variance, mean, variance, covariance and autocorrelation function of the BL-INAR(1) process are given in the following lemma.

**Lemma** **1.** *Let $X_{t}$ be the process in Definition 2. Then it has the following properties:*

*(i) $E\left[X_{t}|X_{t-1}\right]=\alpha X_{t-1}+\mu_{\epsilon }=\alpha X_{t-1}+\theta e^{\theta }$;*

*(ii) $\mathrm{Var}\left[X_{t}|X_{t-1}\right]=\alpha \left(1-\alpha \right)X_{t-1}+\sigma_{\epsilon }^{2}=\alpha \left(1-\alpha \right)X_{t-1}+\theta \left(1+\theta \right)e^{\theta }$;*

*(iii) $\mu :=E\left[X_{t}\right]=\frac{\theta e^{\theta }}{1-\alpha }$;*

*(iv) $\sigma^{2}:=\mathrm{Var}\left[X_{t}\right]=\frac{\theta e^{\theta }\left(1+\alpha +\theta \right)}{1-\alpha^{2}}$;*

*(v) $\gamma_{k}:=\mathrm{Cov}\left(X_{k},X_{k+1}\right)=\alpha^{k}\sigma^{2}$;*

*(vi) $\rho_{k}:=\mathrm{Corr}\left(X_{k},X_{k+1}\right)=\alpha^{k}$.*

The proof of Lemma 1 is similar to that of Theorem 1 of Qi et al. (2019) [14], so it is omitted.

According to Lemma 1, the dispersion index (Fisher, 1950 [27]) of $X_{t}$ is derived as follows:

$$I_{x}:=\frac{\sigma^{2}}{\mu }=1+\frac{\theta }{1+\alpha }>1;$$

thus, the BL-INAR(1) process is suited for overdispersed integer-valued time series.

Additionally, we can obtain the *k*-step ahead conditional mean and *k*-step ahead conditional variance of the BL-INAR(1) process in the following theorem.

**Theorem** **1.** *The k-step ahead conditional mean and k-step ahead conditional variance of the BL-INAR(1) process are given, respectively, by:*

$$E\left(X_{t+k}|X_{t}\right)=\alpha^{k}X_{t}+\mu_{\epsilon }\frac{1-\alpha^{k}}{1-\alpha },$$

*and*

$$\mathrm{Var}\left(X_{t+k}|X_{t}\right)=\alpha^{k}\left(1-\alpha^{k}\right)X_{t}+\mu_{\epsilon }\frac{\left(\alpha -\alpha^{k}\right)\left(1-\alpha^{k}\right)}{1-\alpha^{2}}+\sigma_{\epsilon }^{2}\frac{1-\alpha^{2k}}{1-\alpha^{2}}.$$

For more details about the proof of this theorem, see Qi et al. (2019) [14] and Ristić, Bakouch, and Nastić (2009) [6]. It is easy to see that if k→∞, $E\left(X_{t+k}|X_{t}\right)\to \frac{\mu_{\epsilon }}{1-\alpha }=\frac{\theta e^{\theta }}{1-\alpha }$ and $\mathrm{Var}\left(X_{t+k}|X_{t}\right)\to \frac{\alpha \mu_{\epsilon }+\sigma_{\epsilon }^{2}}{1-\alpha^{2}}=\frac{\theta e^{\theta }\left(1+\alpha +\theta \right)}{1-\alpha^{2}}$, which are the unconditional mean and unconditional variance of $X_{t}$, respectively.

## 3. Estimation of Parameters

The true values of parameters α and θ are unknown in practice; therefore, we need to estimate the value of (α,θ). Sometimes we have to give an estimate of (α,μ) first to get the estimate of (α,θ). In this section, we consider three methods for estimating parameters, namely, CLS, YW and CML.

### 3.1. Conditional Least Squares Estimation

The CLS estimates of the parameters α and θ are obtained by

$$\left(\hat{\alpha },\hat{\theta }\right)=\arg\min\sum_{t=2}^{T}{\left[X_{t}-E\left(X_{t}|X_{t-1}\right)\right]}^{2},$$

and the CLS estimates of (α,μ) are given by

$${\hat{\alpha }}_{\mathrm{CLS}}=\frac{\left(T-1\right)\sum_{t=2}^{T}X_{t}X_{t-1}-\sum_{t=2}^{T}X_{t}\sum_{t=2}^{T}X_{t-1}}{\left(T-1\right)\sum_{t=2}^{T}X_{t-1}^{2}-{\left(\sum_{t=2}^{T}X_{t-1}\right)}^{2}},$$

and

$${\hat{\mu }}_{\mathrm{CLS}}=\frac{\sum_{t=2}^{T}X_{t}-{\hat{\alpha }}_{\mathrm{CLS}}\sum_{t=2}^{T}X_{t-1}}{\left(T-1\right)\left(1-{\hat{\alpha }}_{\mathrm{CLS}}\right)}.$$

Then, the CLS estimate of θ can be obtained by solving the equation ${\hat{\theta }}_{\mathrm{CLS}}e^{{\hat{\theta }}_{\mathrm{CLS}}}={\hat{\mu }}_{\mathrm{CLS}}\left(1-{\hat{\alpha }}_{\mathrm{CLS}}\right)$.

According to Theorems 3.1 and 3.2 in Tjøstheim (1986) [28], we can establish the consistency and asymptotic normality of the CLS estimates ${\hat{\alpha }}_{CLS}$ and ${\hat{\mu }}_{CLS}$ in the following theorem. The proofs of Theorem 2 and the following theorem are given in Appendix A.

**Theorem** **2.** *Let ${\hat{\alpha }}_{CLS}$ and ${\hat{\mu }}_{CLS}$ be the CLS estimates of the BL-INAR(1) process; then ${\left({\hat{\alpha }}_{CLS},\;{\hat{\mu }}_{CLS}\right)}^{\prime }$ is strongly consistent for (α, μ); and the asymptotic distribution follows as:*

$$\sqrt{T}{\left({\hat{\alpha }}_{CLS}-\alpha ,\;{\hat{\mu }}_{CLS}-\mu \right)}^{\prime }\;\overset{d}{\to }\;N\left(0,\Sigma \right),$$

*where*

$$\Sigma =\left[\begin{array}{cc} \frac{\alpha \left(1+\alpha \right)\mu +\sigma_{\epsilon }^{2}}{{\left(1-\alpha \right)}^{2}} & \frac{\alpha \sigma^{2}}{\mu (1+\mu )}\\ \frac{\alpha \sigma^{2}}{\mu (1+\mu )} & \frac{\alpha \left(1-\alpha \right)\left(\mu_{3}-2\mu \sigma^{2}-\mu^{3}\right)+\sigma_{\epsilon }^{2}\sigma^{2}}{\mu^{2}{\left(1+\mu \right)}^{2}} \end{array}\right],$$

*and $\mu_{3}=E\left(X_{t}^{3}\right)=\frac{\left(1-\alpha^{3}\right)\mu^{3}+\left(1+2\alpha^{2}-3\alpha^{3}\right)\mu \sigma^{2}+\alpha^{2}\left(1-\alpha \right)\sigma^{2}}{1-\alpha^{3}}$.*

Using the delta method, we can obtain the limit distribution of $\left(\hat{\alpha },\hat{\theta }\right)$, and we can also know that $\hat{\theta }$ is consistent.

### 3.2. Yule–Walker Estimation

Let $X_{1},\ldots ,X_{T}$ come from the process $\left\{X_{t}\right\}$ in Definition 2. The sample mean is $\bar{X}=\frac{1}{T}\sum_{t=1}^{T}X_{t}$, and the sample autocorrelation function is

$${\hat{\rho }}_{k}=\frac{\sum_{t=1}^{T-k}\left(X_{t}-\bar{X}\right)\left(X_{t+k}-\bar{X}\right)}{\sum_{t=1}^{n}{\left(X_{t}-\bar{X}\right)}^{2}}.$$

From Lemma 1, we know $\rho_{k}=\alpha^{k}$, thus the Yule–Walker (YW) estimate of α is given by

$${\hat{\alpha }}_{\mathrm{YW}}=\hat{\rho }\left(1\right)=\frac{\sum_{t=1}^{T-1}\left(X_{t}-\bar{X}\right)\left(X_{t+1}-\bar{X}\right)}{\sum_{t=1}^{T}{\left(X_{t}-\bar{X}\right)}^{2}},$$

and

$${\hat{\mu }}_{\mathrm{YW}}=\bar{X},$$

with $\mu =\frac{\theta e^{\theta }}{1-\alpha }$; then the estimate of θ can be obtained.

For asymptotic properties of the YW estimates, Freeland and McCabe (2005) [29] showed that the YW and CLS estimates are asymptotically equivalent for a Poisson INAR(1) process. The next theorem shows that the conclusion holds for our BL-INAR(1) process.

**Theorem** **3.** *In the BL-INAR(1) process, CLS and YW estimates are asymptotically equivalent, i.e.,*

$${\hat{\alpha }}_{\mathrm{CLS}}-{\hat{\alpha }}_{\mathrm{YW}}=o_{p}\left(T^{-\frac{1}{2}}\right)\;\;\;\mathrm{and}\;\;\;{\hat{\theta }}_{\mathrm{CLS}}-{\hat{\theta }}_{\mathrm{YW}}=o_{p}\left(T^{-\frac{1}{2}}\right).$$

### 3.3. Conditional Maximum Likelihood Estimation

According to the joint probability function (6), the likelihood function can be obtained as:

$$\begin{array}{cc} f(x_{1},x_{2},\ldots ,x_{T}) & =P\left(X_{1}=x_{1}\right)\prod_{t=1}^{T-1}P\left(X_{t+1}=x_{t+1}|X_{t}=x_{t}\right)\\ \; & =f\left(x_{1}\right)\prod_{t=1}^{T-1}\left[\sum_{m=0}^{\min\left(x_{t},x_{t+1}\right)}\left(\begin{array}{c} x_{t}\\ m \end{array}\right)\alpha^{m}{\left(1-\alpha \right)}^{x_{t}-m}P\left(\epsilon_{t+1}=x_{t+1}-m\right)\right]. \end{array}$$

To condition on variable $X_{1}$, we can obtain the conditional log likelihood function as:

$$L\left(\alpha ,\theta \right)=\sum_{t=1}^{T-1}\log P\left(X_{t+1}=x_{t+1}|X_{t}=x_{t}\right),$$

the CML estimates of (α,θ) are the values of $\left({\hat{\alpha }}_{\mathrm{CML}},{\hat{\theta }}_{\mathrm{CML}}\right)$ obtained by maximizing the conditional log likelihood function L(α,θ). It is easy to check that the BL-INAR(1) process satisfies conditions (C1)–(C6) of Franke and Seligmann (1993) [30]; thus, the CML estimates $\left({\hat{\alpha }}_{\mathrm{CML}},{\hat{\theta }}_{\mathrm{CML}}\right)$ are consistent and asymptotically normal. The proof is similar to those of Theorems 22.4 and 22.5 of Franke and Seligmann (1993) [30], so it is omitted.

## 4. Simulation

A Monte Carlo simulation was conducted to study the performances of the CLS, YW, and CML estimates of the BL-INAR(1) model. The CML estimates were obtained by using the BFGS quasi-Newton nonlinear optimization algorithm with numerical derivatives. We considered YW estimates as initial values for the algorithm. The simulation was conducted using R programming language, and the size of the sample was 100, 250, 500, or 1000. The number of replicates was 1000. For the true values of parameters, we considered α=0.25,0.5, and 0.75 and θ=0.5, and 1.5.

**Figure 1:**
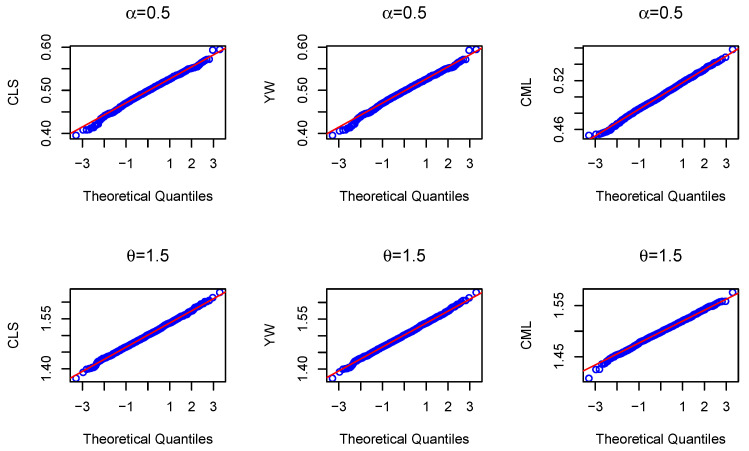
The Q–Q plots of the CLS, YW, and CML estimates for the BL-INAR(1) model with sample size T=1000.

First, we give the Q–Q plots of the CLS, YW, and CML estimates for the BL-INAR(1) model with sample size T=1000, α=0.5, and θ=1.5 in Figure 1. From the six Q–Q plots, we can see that they contain roughly straight lines; i.e., the estimates of the parameters are normally distributed. Then, the numerical simulation results are presented in Table 1 and Table 2. By comparing the two tables, we can find that with the same θ and *T*, the mean squared error (MSE) for the estimate of θ increased with the increase of α, but the MSE for the estimate of α decreased. Additionally, the MSE for the estimate of θ increased with the increase of θ with the same α and *T*, but the MSE for the estimate of α decreased. Furthermore, we can observe that the estimates of CLS and YW are similar, and the bias tended toward zero for all estimates as the sample size increased. The estimates of CML converged faster to the true parameter values. We conclude that the CML estimates produced the smallest mean square errors, and CML performed better than CLS and YW.

## 5. Real Data Examples

In this section, we present two applications of the BL-INAR(1) model to real datasets, and compare it with the P-INAR(1), G-INAR(1), PL-INAR(1), NB-INAR(1), ZIP-INAR(1), DP-INAR(1), and GP-INAR(1) models. Results of the comparison are discussed here as well.

### 5.1. Disconduct Data

The first dataset is a monthly count of disconduct in the first census tract in Rochester, which can be obtained from Available online: http://www.forecastingprinciples.com (accessed on 8 May 2012). The data comprise 132 observations (T=132) starting from January 1991 and ending in December 2001.

**Figure 2:**
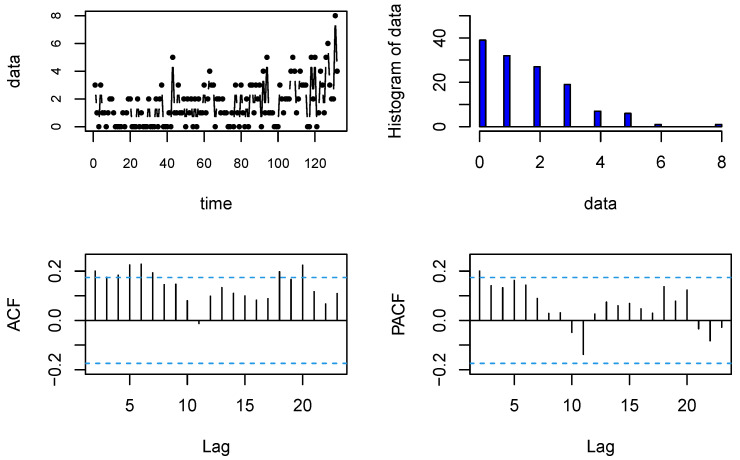
The time plot, histogram, ACF, and PACF of disconduct data.

The time plot, histogram, autocorrelation function (ACF), and partial autocorrelation function (PACF) are provided in Figure 2. We applied the Ljung–Box test (Ljung and Box (1978) [31]) to check whether this time series dataset has any autocorrelation. The *p*-value of the Ljung–Box test is $1.317\times 10^{-5}$, which is less than 0.05. This means that the time series data have some autocorrelation, and according to the PACF diagram, the data are first-order autocorrelated, which shows that the AR(1)-type process is appropriate for modeling this dataset.

The sample mean and variance of the data are $\bar{X}=1.6288$ and $S_{X}^{2}=2.4455$, respectively. Thus, we got the dispersion index ${\hat{I}}_{x}=S_{X}^{2}/\bar{X}=1.5014$. According to the overdispersion test of Schweer and Weiß (2014) [11], the critical value of the data is 1.1994. The dispersion index ${\hat{I}}_{x}$ exceeds the critical value, which means that the equidispersed P-INAR(1) model is not a good choice for the data.

For comparison, we calculated the CML estimates of parameters, and the AIC, BIC, CAIC, HQIC, fitted mean, and fitted variance of the BL-INAR(1) model, the P-INAR(1) model, the G-INAR (1) model, the PL-INAR(1) model, the ZIP-INAR(1) model, the NB-INAR(1) model, the DP-INAR(1) model, and the GP-INAR(1) model. Among the eight models, the first four are two-parameter models and the last four are three-parameter models. The results are presented in Table 3. We found that the AIC, BIC, CAIC, and HQIC of the BL-INAR(1) model were smaller than those of others. We also found that the fitted means of all eight models were near to the sample mean, and the fitted mean of the PL-INAR(1) model was the closest to the sample mean. In terms of fitted variance, Table 3 shows that the fitted variance of the BL-INAR(1) model performed better than those of the other seven models.

**Figure 3:**
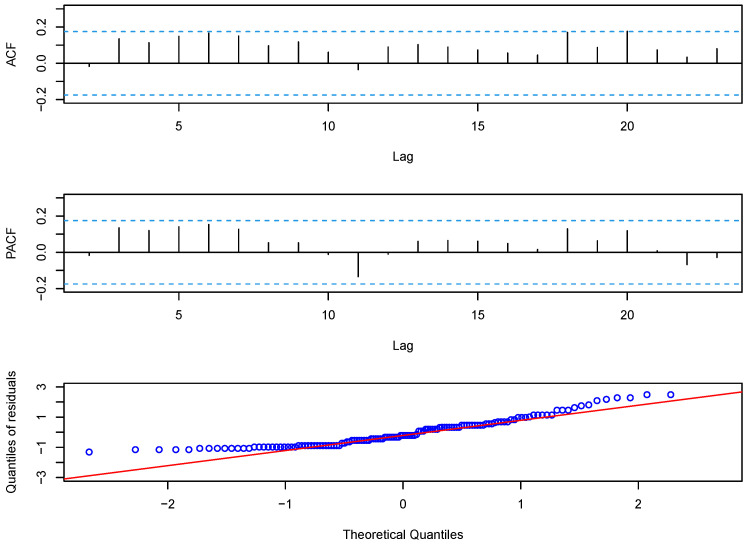
The ACF, PACF, and Q–Q plots of the Pearson residual for disconduct data using the BL-INAR(1) model.

For the prediction, we used the first 126 observations to estimate the parameters, and then predicted the last six observations. The predicted values of the disconduct data could be given by $E\left(X_{t+k}|X_{t}\right)=\alpha^{k}X_{t}+\mu_{\epsilon }\frac{1-\alpha^{k}}{1-\alpha }$. For a further comparison of models, we calculated the root mean square values of the prediction errors (RMSEs) for the last 6 months of the data, and the RMSE is defined as $\mathrm{RMSE}=\sqrt{\frac{1}{6}\sum_{k=1}^{6}{\left(X_{t+k}-{\hat{X}}_{t+k}\right)}^{2}}$. We present the RMSE results of eight models in the last column of Table 3. From the table, we can see that the RMSE of the G-INAR(1) model was best. The RMSE of the BL-INAR(1) model is smaller than those of the P-INAR(1) model, the NB-INAR(1) model, the DP-INAR(1) model, and the GP-INAR(1) model; and a little larger than those of the G-INAR(1) model, the PL-INAR(1) model, and the ZIP-INAR(1) model. Although the fitted mean and RMSE of the BL-INAR(1) model are not the best, it is the best choice under the other five criteria. Further, we analyze the Pearson residuals, and Figure 3 plots the ACF, PACF, and Q–Q plots of residuals. The ACF and PACF graphs show no correlation between residuals, which is supported by the result of the Ljung–Box test with a *p*-value of 0.05251>0.05. The Q–Q plots appear to be roughly normally distributed, as we expected. Hence, we can conclude that the BL-INAR(1) model is the most suitable among those available for this dataset.

### 5.2. Strikes Data

**Figure 4:**
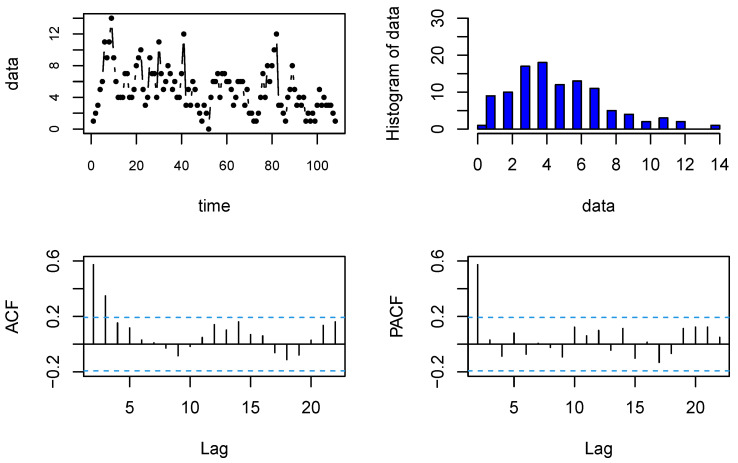
The time plot, histogram, ACF, and PACF of data on strikes.

The second dataset, which was analyzed by Weiß (2010) [32], is the monthly number of work stoppages (strikes and lock-outs) of 1000 or more workers for the period 1994–2002. It was published by the US Bureau of Labor Statistics and can be obtained by online at the address Available online: http://www.bls.gov/wsp/ (accessed on 8 May 2012). The data contain 108 observations, and the time plot, histogram, ACF, and PACF are provided in Figure 4. As with the previous example, the Ljung–Box test was used to check whether the strike data have any autocorrelation. The *p*-value of the Ljung–Box test was $2.372\times 10^{-8}$, which shows that the time series data have some autocorrelation, and according to the PACF diagram, it is also first-order autocorrelated, so an AR(1)-type process is appropriate for modeling this dataset.

**Figure 5:**
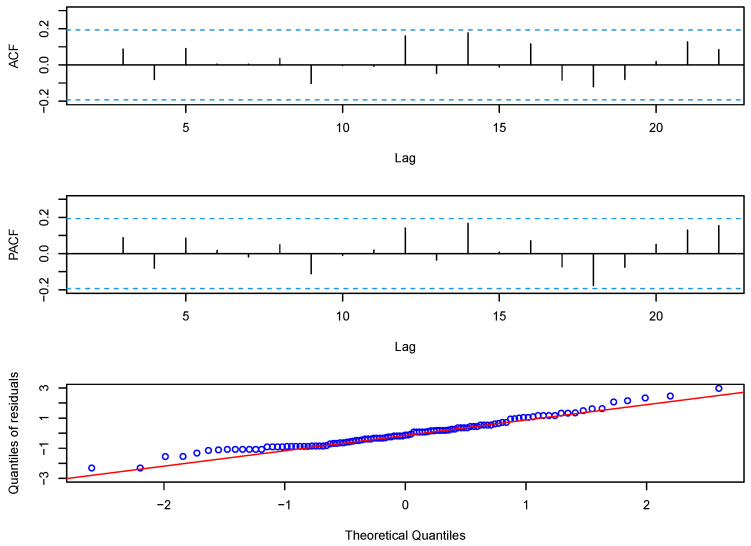
The ACF, PACF, and Q–Q plots of the Pearson residual for strike data using the BL-INAR(1) model.

The sample mean, variance, and dispersion index were calculated to be 4.9444, 7.8488, and 1.5874, respectively. According to the overdispersion test, the critical value of the data is 1.2808, and we observe that it was inappropriate to use the P-INAR(1) model to fit the data. The CML estimates, AIC, BIC, CAIC, HQIC, fitted mean, and fitted variance for the BL-INAR(1), P-INAR(1), G-INAR(1), PL-INAR(1), NB-INAR(1), ZIP-INAR(1), DP-INAR(1), and GP-INAR(1) models were obtained and are shown in Table 4. We see that the AIC, BIC, CAIC, and HQIC of the BL-INAR(1) model are smaller than those of others, and the fitted mean of the BL-INAR(1) model is not much different from those of the other seven models. Further, we can see that the BL-INAR(1) model performed better than others when calculating the fitted variance. Similarly to the previous example, the first 102 observations were used to estimate the parameters and predict the last six observations. The RMSE of the predictions is also presented in Table 4. We can observe that the RMSE of the G-INAR(1) model is the smallest; however, it is only 0.05 less than the RMSE of the BL-INAR(1) model. As in the previous example, although the BL-INAR(1) model was not the best under the fitted mean and RMSE criteria, it performed best under the other five criteria. Additionally, we show the Pearson residuals analysis. Figure 5 gives the ACF, PACF, and Q–Q plots of the residuals. We found that there is no evidence of any significant correlation within the residuals, a finding also supported by the Ljung–Box test with a *p*-value of 0.9522, which is greater than 0.05. The Q–Q plot also appears to be roughly normally distributed. Thus, according to above discussions and its simplicity, we can conclude that the BL-INAR(1) model was the most appropriate.

Combined with the above two examples and the advantages of the Bell distribution with one parameter and a simple form, the BL-INAR(1) model is competitive with the other seven models.

## 6. Conclusions

A new INAR(1) model with Bell innovations based on the binomial thinning operator was introduced in this paper. Based on the overdispersed property of the Bell distribution, we found that the BL-INAR(1) model is suitable for overdispersed data. Some basic properties of the model were obtained, such as transition probabilities, conditional mean, conditional variance, mean, variance, covariance, autocorrelation function, and *k*-step ahead conditional mean and variance. For unknown parameters, CLS, YW, and CML methods are used to estimate them. The Q–Q plots showed that the estimates of the parameters are normally distributed. The simulated results revealed that the CML estimates of parameters of the BL-INAR(1) model were better than the CLS and YW estimates. Finally, by comparing the AIC values, BIC values, CAIC values, HQIC values, fitted means, fitted variances, and RMSE values of the predictions among eight INAR(1) models, two real datasets both showed that the BL-INAR(1) model fits better than other INAR(1) models. The analysis of residuals also shows that the BL-INAR(1) model provided adequate fits to those datasets.

Although there are many overdispersed INAR(1) models, some interesting properties of the Bell distribution, such as having one parameter, infinitely divisibility, having a simple probability mass function, belonging to the one-parameter exponential family of distributions, and for a parameter with a small value, having the Bell distribution approach the Poisson distribution, make the BL-INAR(1) model competitive. Some extended distributions of the Bell distribution, such as the zero-inflated Bell distribution and the Bell–Touchard distribution, provide ideas for us to study related INAR models in the future.

## Figures and Tables

**Table 1:** Empirical means and mean squared errors (in parentheses) of the estimates of the parameters for some values of α and θ of the BL-INAR(1) model.

*T*	${\hat{\alpha }}_{\mathrm{CLS}}$	${\hat{\theta }}_{\mathrm{CLS}}$	${\hat{\alpha }}_{\mathrm{YW}}$	${\hat{\theta }}_{\mathrm{YW}}$	${\hat{\alpha }}_{\mathrm{CML}}$	${\hat{\theta }}_{\mathrm{CML}}$
(α, θ) = (0.25, 0.5)						
100	0.220445	0.507901	0.218464	0.508838	0.238497	0.500617
	(0.011615)	(0.003871)	(0.011564)	(0.003814)	(0.007120)	(0.003049)
250	0.240011	0.502610	0.239107	0.503039	0.246433	0.500099
	(0.004665)	(0.001520)	(0.004653)	(0.001501)	(0.002658)	(0.001146)
500	0.245254	0.500707	0.244710	0.500965	0.247766	0.499777
	(0.002286)	(0.000758)	(0.002284)	(0.000759)	(0.001300)	(0.000603)
1000	0.246458	0.500868	0.246197	0.500974	0.249120	0.499768
	(0.001195)	(0.000379)	(0.001197)	(0.000380)	(0.000714)	(0.000290)
(α, θ) = (0.5, 0.5)						
100	0.475430	0.508229	0.469977	0.512782	0.495566	0.497046
	(0.010198)	(0.005396)	(0.010256)	(0.005296)	(0.004046)	(0.003083)
250	0.488517	0.504259	0.486491	0.505890	0.497636	0.499045
	(0.003895)	(0.002128)	(0.003911)	(0.002123)	(0.001723)	(0.001332)
500	0.493426	0.502160	0.492388	0.503029	0.498222	0.499355
	(0.001857)	(0.001026)	(0.001868)	(0.001025)	(0.000866)	(0.000643)
1000	0.496426	0.501635	0.495922	0.502043	0.499262	0.499936
	(0.000914)	(0.000530)	(0.000916)	(0.000529)	(0.000412)	(0.000322)
(α, θ) = (0.75, 0.5)						
100	0.714977	0.535092	0.707308	0.543643	0.745993	0.500460
	(0.006966)	(0.011276)	(0.007639)	(0.011838)	(0.001321)	(0.003355)
250	0.736256	0.513360	0.733057	0.517222	0.748974	0.498915
	(0.002354)	(0.004357)	(0.002456)	(0.004432)	(0.000494)	(0.001352)
500	0.743674	0.505799	0.742084	0.507695	0.749245	0.499568
	(0.001052)	(0.001967)	(0.001079)	(0.001983)	(0.000243)	(0.000681)
1000	0.746006	0.504828	0.745283	0.505726	0.749925	0.500221
	(0.000546)	(0.001001)	(0.000554)	(0.001011)	(0.000132)	(0.000309)

**Table 2:** Empirical means and mean squared errors (in parentheses) of the estimates of the parameters for some values of α and θ of the BL-INAR(1) model.

*T*	${\hat{\alpha }}_{\mathrm{CLS}}$	${\hat{\theta }}_{\mathrm{CLS}}$	${\hat{\alpha }}_{\mathrm{YW}}$	${\hat{\theta }}_{\mathrm{YW}}$	${\hat{\alpha }}_{\mathrm{CML}}$	${\hat{\theta }}_{\mathrm{CML}}$
(α, θ) = (0.25, 1.5)						
100	0.230059	1.508538	0.227601	1.510489	0.252786	1.492707
	(0.010409)	(0.007500)	(0.010294)	(0.007323)	(0.004877)	(0.004375)
250	0.243290	1.503077	0.242313	1.503896	0.250278	1.498450
	(0.003994)	(0.002928)	(0.003976)	(0.002898)	(0.001810)	(0.001660)
500	0.244804	1.503143	0.244310	1.503531	0.249992	1.499429
	(0.001917)	(0.001459)	(0.001914)	(0.001451)	(0.000913)	(0.000829)
1000	0.248715	1.500420	0.248470	1.500628	0.251744	1.498222
	(0.000984)	(0.000745)	(0.000983)	(0.000745)	(0.000477)	(0.000422)
(α, θ) = (0.5, 1.5)						
100	0.472192	1.522593	0.467254	1.528069	0.497401	1.497773
	(0.008714)	(0.011950)	(0.008913)	(0.011884)	(0.002653)	(0.004999)
250	0.489125	1.509054	0.487244	1.511225	0.499745	1.498361
	(0.003127)	(0.004609)	(0.003148)	(0.004598)	(0.000991)	(0.001856)
500	0.496116	1.502407	0.495032	1.503670	0.501865	1.496562
	(0.001650)	(0.002493)	(0.001660)	(0.002487)	(0.000584)	(0.001078)
1000	0.497904	1.501246	0.497432	1.501800	0.500976	1.498100
	(0.000826)	(0.001314)	(0.000827)	(0.001314)	(0.000274)	(0.000502)
(α, θ) = (0.75, 1.5)						
100	0.721350	1.547389	0.713291	1.565523	0.749555	1.495159
	(0.005627)	(0.025790)	(0.006188)	(0.026764)	(0.000827)	(0.005581)
250	0.736975	1.522062	0.733930	1.529286	0.749880	1.497782
	(0.002181)	(0.011299)	(0.002278)	(0.011488)	(0.000343)	(0.002363)
500	0.742692	1.512717	0.741144	1.516338	0.749888	1.498329
	(0.000919)	(0.005007)	(0.000947)	(0.005076)	(0.000158)	(0.001101)
1000	0.747785	1.503485	0.747046	1.505296	0.750224	1.499187
	(0.000476)	(0.002670)	(0.000479)	(0.002670)	(0.000083)	(0.000541)

**Table 3:** CML estimates, AIC, BIC, CAIC, HQIC, fitted mean, fitted variance, and RMSE for eight INAR(1) models of disconduct data.

Model	Parameters	AIC	BIC	CAIC	HQIC	Mean	Variance	RMSE
BL-INAR	$\hat{\alpha }=0.1882$	**441.7380** ${}^{1}$	**447.5036** ${}^{1}$	**449.5036** ${}^{1}$	**444.0809** ${}^{1}$	1.6201	**2.5361** ${}^{2}$	3.2205
	$\hat{\theta }=0.6718$							
P-INAR	$\hat{\alpha }=0.1496$	456.4653	462.2309	464.2309	458.8082	1.6197	2.1512	3.2497
	$\hat{\lambda }=1.3773$							
G-INAR	$\hat{\alpha }=0.2405$	446.1416	451.9072	453.9072	448.4845	1.6207	3.2290	**3.1803** ${}^{1}$
	$\hat{\pi }=0.4482$							
PL-INAR	$\hat{\alpha }=0.2197$	444.3542	450.1198	452.1198	446.6971	**1.6254** ${}^{2}$	0.2928	3.1929
	$\hat{\theta }=1.1545$							
NB-INAR	$\hat{\alpha }=0.1845$	445.4351	454.0835	457.0835	448.9494	1.6201	2.5542	3.2233
	$\hat{n}=1.9345$							
	$\hat{\pi }=0.5942$							
ZIP-INAR	$\hat{\alpha }=0.1992$	442.7224	451.3708	454.3708	446.2367	1.6202	2.3903	3.2121
	$\hat{\lambda }=1.8674$							
	$\hat{\rho }=0.3052$							
DP-INAR	$\hat{\alpha }=0.1865$	443.7622	452.4106	455.4106	447.2765	1.4900	2.6859	3.3155
	$\hat{\mu }=1.2121$							
	$\hat{\phi }=0.5122$							
GP-INAR	$\hat{\alpha }=0.1820$	445.8156	454.4640	457.4640	449.3300	1.6200	2.5386	3.2252
	$\hat{\mu }=1.0254$							
	$\hat{\phi }=0.2262$							

${}^{1}$ Bold text means the smallest value in the column. ${}^{2}$ Bold text means that this value is the closest in the column to the sample value described in the text.

**Table 4:** CML estimates, AIC, BIC, CAIC, HQIC, fitted mean, fitted variance, and RMSE from eight INAR(1) models of strike data.

Model	Parameters	AIC	BIC	CAIC	HQIC	Mean	Variance	RMSE
BL-INAR	$\hat{\alpha }=0.5789$	**468.1557** ${}^{1}$	**473.5199** ${}^{1}$	**475.5199** ${}^{1}$	**470.3307** ${}^{1}$	4.9813	**7.7408** ${}^{2}$	2.2659
	$\hat{\theta }=0.8747$						
P-INAR	$\hat{\alpha }=0.5061$	473.0936	478.4578	480.4578	475.2686	4.9813	9.8110	2.3331
	$\hat{\lambda }=2.4603$						
G-INAR	$\hat{\alpha }=0.6235$	475.3209	480.6852	482.6852	477.4960	4.9813	10.7361	**2.2121** ${}^{1}$
	$\hat{\pi }=0.3478$							
PL-INAR	$\hat{\alpha }=0.6062$	471.9345	477.2987	479.2987	474.1095	5.0016	1.8876	2.2489
	$\hat{\theta }=0.7911$						
NB-INAR	$\hat{\alpha }=0.5483$	469.6850	477.7314	480.7314	472.9476	4.9813	6.8573	2.2969
	$\hat{n}=3.8582$							
	$\hat{\pi }=0.6317$							
ZIP-INAR	$\hat{\alpha }=0.5785$	470.9985	479.0449	482.0449	474.2610	4.9813	6.6692	2.2663
	$\hat{\lambda }=2.6343$						
	$\hat{\rho }=0.2030$						
DP-INAR	$\hat{\alpha }=0.5617$	469.5585	477.6048	480.6048	472.8210	**4.9576** ${}^{2}$	7.1420	2.2659
	$\hat{\mu }=2.1727$						
	$\hat{\phi }=0.5924$						
GP-INAR	$\hat{\alpha }=0.5464$	469.7467	477.7930	480.7930	473.0092	4.9813	6.8335	2.2986
	$\hat{\mu }=1.8003$						
	$\hat{\phi }=0.2032$						

${}^{1}$ Bold means the smallest value in the column. ${}^{2}$ Bold means that this value is the closest in the column to the sample value described in the text.

## Data Availability

The disconduct data and the strike data are available at http://www.forecastingprinciples.com (accessed on 1 June 2021) and http://www.bls.gov/wsp/ (accessed on 1 June 2021 ), respectively .

## Appendix A

### Appendix A.1. Proof of Theorem 2

To prove this theorem, we need to show that the conditions given in Theorems 3.1 and 3.2 of Tjøstheim (1986) [28] are satisfied.

Define ϕ=(α,μ), and the true value of the unknown parameter $\phi_{0}=\left(\alpha_{0},\mu_{0}\right)$. According to Lemma 1, we know that $E[X_{t}^{2}]<\infty$ and that $E[X_{t}|X_{t-1}]$ is almost surely three times differentiable in an open set Φ containing $\phi_{0}$.

Condition 1:

$E\left\{{\left|\frac{\partial E\left(X_{t}|X_{t-1}\right)}{\partial \phi_{i}}\left(\phi_{0}\right)\right|}^{2}\right\}<\infty$ and $E\left\{{\left|\frac{\partial^{2}E\left(X_{t}|X_{t-1}\right)}{\partial \phi_{i}\partial \phi_{j}}\left(\phi_{0}\right)\right|}^{2}\right\}<\infty$, for i,j=1,2.

According to $E\left(X_{t}|X_{t-1}\right)=\alpha X_{t-1}+\left(1-\alpha \right)\mu$, we have

$$\begin{array}{c} E\left\{{\left|\frac{\partial E\left(X_{t}|X_{t-1}\right)}{\partial \alpha }\left(\phi_{0}\right)\right|}^{2}\right\}=E\left\{{\left|X_{t-1}-\mu_{0}\right|}^{2}\right\}=\mathrm{Var}\left(X_{t-1}\right)<\infty \;\mathrm{and}\;\\ E\left\{{\left|\frac{\partial E\left(X_{t}|X_{t-1}\right)}{\partial \mu }\left(\phi_{0}\right)\right|}^{2}\right\}=E\left\{{\left|1-\alpha_{0}\right|}^{2}\right\}={\left(1-\alpha_{0}\right)}^{2}<\infty . \end{array}$$

For the second derivative of $E\left(X_{t}|X_{t-1}\right)$, we have

$$\begin{array}{c} E\left\{{\left|\frac{\partial^{2}E\left(X_{t}|X_{t-1}\right)}{\partial \alpha^{2}}\left(\phi_{0}\right)\right|}^{2}\right\}=E\left\{{\left|\frac{\partial^{2}E\left(X_{t}|X_{t-1}\right)}{\partial \mu^{2}}\left(\phi_{0}\right)\right|}^{2}\right\}=0<\infty \;\mathrm{and}\;\\ E\left\{{\left|\frac{\partial^{2}E\left(X_{t}|X_{t-1}\right)}{\partial \alpha \partial \mu }\left(\phi_{0}\right)\right|}^{2}\right\}=1<\infty . \end{array}$$

Condition 2:

The vectors $\partial E\left(X_{t}|X_{t-1}\right)\left(\theta_{0}\right)/\partial \phi_{i},i,j=1,2$ are linearly independent in the sense that if $a_{1}$ and $a_{2}$ are arbitrary real numbers such that

$$E\left\{{\left|\sum_{i=1}^{2}a_{i}\frac{\partial E\left(X_{t}|X_{t-1}\right)}{\partial \phi_{i}}\left(\phi_{0}\right)\right|}^{2}\right\}=0,$$

then $a_{1}=a_{2}=0$. Note that

$$\begin{array}{cc} E\left\{{\left|a_{1}\frac{\partial E\left(X_{t}|X_{t-1}\right)}{\partial \alpha }\left(\phi_{0}\right)+a_{2}\frac{\partial E\left(X_{t}|X_{t-1}\right)}{\partial \mu }\left(\phi_{0}\right)\right|}^{2}\right\} & =0\Rightarrow \\ E\left\{{\left|a_{1}\left(X_{t-1}-\mu_{0}\right)+a_{2}\left(1-\alpha_{0}\right)\right|}^{2}\right\} & =0\Rightarrow \\ \underset{\geq 0}{\underset{︸}{a_{1}^{2}\mathrm{Var}\left(X_{t-1}\right)}}+\underset{\geq 0}{\underset{︸}{a_{2}^{2}{\left(1-\alpha_{0}\right)}^{2}}} & =0\Rightarrow \\ a_{1}^{2}\underset{>0}{\underset{︸}{\mathrm{Var}\left(X_{t-1}\right)}}=0\;\mathrm{and}\;a_{2}^{2}\underset{>0}{\underset{︸}{{\left(1-\alpha_{0}\right)}^{2}}} & =0\Rightarrow \\ a_{1}^{2}=0\;\mathrm{and}\;a_{2}^{2} & =0. \end{array}$$

Then $a_{1}=a_{2}=0$.

Condition 3:

For ϕ∈Φ, there exist functions $G_{t-1}^{ijk}\left(X_{1},\ldots ,X_{t-1}\right)$ and $H_{t}^{ijk}\left(X_{1},\ldots ,X_{t}\right)$ for i,j=1,2 such that

$$\begin{array}{cc} M_{t-1}^{ijk}\left(\phi \right) & =\left|\frac{\partial E\left(X_{t}|X_{t-1}\right)}{\partial \phi_{i}}\left(\phi \right)\frac{\partial^{2}E\left(X_{t}|X_{t-1}\right)}{\partial \phi_{j}\partial \phi_{k}}\left(\phi \right)\right|\leq G_{t-1}^{ijk},\;E\left(G_{t-1}^{ijk}\right)<\infty ,\\ N_{t}^{ijk}\left(\phi \right) & =\left|\left\{X_{t}-E\left(X_{t}|X_{t-1}\right)\left(\phi \right)\right\}\frac{\partial^{3}E\left(X_{t}|X_{t-1}\right)}{\partial \phi_{i}\partial \phi_{j}\partial \phi_{k}}\left(\phi \right)\right|\leq H_{t}^{ijk},\;E\left(H_{t}^{ijk}\right)<\infty . \end{array}$$

Note that $M_{t-1}^{111}\left(\phi \right)=M_{t-1}^{122}\left(\phi \right)=M_{t-1}^{211}\left(\phi \right)=M_{t-1}^{222}\left(\phi \right)=0$ and

$$\begin{array}{cc} M_{t-1}^{112}\left(\phi \right) & =M_{t-1}^{121}\left(\phi \right)=\left|X_{t-1}-\mu \right|,\\ M_{t-1}^{212}\left(\phi \right) & =M_{t-1}^{221}\left(\phi \right)=\left|\alpha -1\right|<1; \end{array}$$

then we can choose $G_{t-1}^{ijk}\left(\phi \right)={\left(X_{t-1}-\mu \right)}^{2}+1,\forall i,j,k=1,2$, which guarantees that $M_{t-1}^{ijk}\left(\phi \right)<G_{t-1}^{ijk}\left(\phi \right)$ and $E\left(G_{t-1}^{ijk}\right)=\mathrm{Var}\left(X_{t-1}+1\right)<\infty$.

For $N_{t}^{ijk}\left(\phi \right)$, it is easy to know that $N_{t}^{ijk}\left(\phi \right)=0,\forall i,j,k=1,2$. So we choose $H_{t}^{ijk}\left(\phi \right)=0$, ∀i,j,k=1,2 to satisfy $N_{t}^{ijk}\left(\phi \right)<H_{t}^{ijk}\left(\phi \right)$ and $E(H_{t}^{ijk})=0<\infty$.

The above three conditions ensure that $\left({\hat{\alpha }}_{CLS},{\hat{\mu }}_{CLS}\right)$ is a strongly consistent estimator for (α,μ). According to Theorem 3.2 in Tjøstheim (1986) [28], the asymptotic distribution of $\left({\hat{\alpha }}_{CLS},{\hat{\mu }}_{CLS}\right)$ is

$$\sqrt{T}{\left({\hat{\alpha }}_{CLS}-\alpha ,\;{\hat{\mu }}_{CLS}-\mu \right)}^{\prime }\;\overset{d}{\to }\;N\left(0,\Sigma \right),$$

where $\Sigma =U^{-1}RU^{-1}$,

$$\begin{array}{cc} U & =E\left\{\frac{\partial E{\left(X_{t}|X_{t-1}\right)}^{T}}{\partial \phi }\left(\phi \right)\cdot \frac{\partial E\left(X_{t}|X_{t-1}\right)}{\partial \phi }\left(\phi \right)\right\},\\ R & =E\left\{\frac{\partial E{\left(X_{t}|X_{t-1}\right)}^{T}}{\partial \phi }\left(\phi \right)f_{t|t-1}\left(\phi \right)\frac{\partial E\left(X_{t}|X_{t-1}\right)}{\partial \phi }\left(\phi \right)\right\}, \end{array}$$

and

$$f_{t|t-1}\left(\phi \right)=E\left\{\left(X_{t}-E\left(X_{t}|X_{t-1}\right)\right){\left(X_{t}-E\left(X_{t}|X_{t-1}\right)\right)}^{T}|X_{t-1}\right\}.$$

We can then find that

$$\Sigma =\left[\begin{array}{cc} \frac{\alpha \left(1+\alpha \right)\mu +\sigma_{\epsilon }^{2}}{{\left(1-\alpha \right)}^{2}} & \frac{\alpha \sigma^{2}}{\mu (1+\mu )}\\ \frac{\alpha \sigma^{2}}{\mu (1+\mu )} & \frac{\alpha \left(1-\alpha \right)\left(\mu_{3}-2\mu \sigma^{2}-\mu^{3}\right)+\sigma_{\epsilon }^{2}\sigma^{2}}{\mu^{2}{\left(1+\mu \right)}^{2}} \end{array}\right],$$

where $\mu_{3}=E\left(X_{t}^{3}\right)=\frac{\left(1-\alpha^{3}\right)\mu^{3}+\left(1+2\alpha^{2}-3\alpha^{3}\right)\mu \sigma^{2}+\alpha^{2}\left(1-\alpha \right)\sigma^{2}}{1-\alpha^{3}}$, which follows from the following derivation:

$$\begin{array}{cc} \mu_{3}= & E\left[X_{t}^{3}\right]=E\left[X_{t}^{2}\left(\alpha \circ X_{t-1}+\epsilon_{t}\right)\right]=E\left[E\left[X_{t}^{2}\left(\alpha \circ X_{t-1}+\epsilon_{t}\right)|X_{t-1}\right]\right]\\ = & E[\alpha X_{t-1}E\left[X_{t}^{2}|X_{t-1}\right]+\mu_{\epsilon }E\left[X_{t}^{2}|X_{t-1}\right]]\\ = & E\left[\alpha X_{t-1}E\left[X_{t}^{2}|X_{t-1}\right]\right]+\mu_{\epsilon }E\left[X_{t}^{2}\right], \end{array}$$

according to Lemma 1,

$$\begin{array}{cc} E[X_{t}^{2}|X_{t-1}]= & \mathrm{Var}\left[X_{t}|X_{t-1}\right]+{\left(E\left[X_{t}|X_{t-1}\right]\right)}^{2}\\ = & \alpha^{2}X_{t}^{2}+\left(\alpha \left(1-\alpha \right)+2\alpha \mu_{\epsilon }\right)X_{t}+\mu_{\epsilon }^{2}+\sigma_{\epsilon }^{2}; \end{array}$$

then, we have

$$\begin{array}{cc} \mu_{3}= & E\left[\alpha X_{t-1}E\left[X_{t}^{2}|X_{t-1}\right]\right]+\mu_{\epsilon }E\left[X_{t}^{2}\right]\\ = & \alpha^{3}E\left[X_{t-1}^{3}\right]+\alpha^{2}\left(1-\alpha \right)E\left[X_{t-1}^{2}\right]+2\alpha^{2}\mu_{\epsilon }E\left[X_{t-1}^{2}\right]+\alpha \mu \left(\mu_{\epsilon }^{2}+\sigma_{\epsilon }^{2}\right)+\mu_{\epsilon }E\left[X_{t}^{2}\right]\\ \; & \left(\mathrm{where}\;\mu_{\epsilon }=\left(1-\alpha \right)\mu \;\mathrm{and}\;\sigma_{\epsilon }^{2}=\left(1-\alpha^{2}\right)\sigma^{2}-\alpha \left(1-\alpha \right)\mu \right)\\ = & \alpha^{3}\mu_{3}+\left(1-\alpha^{3}\right)\mu^{3}+\left(1+2\alpha^{2}-3\alpha^{3}\right)\mu \sigma^{2}+\alpha^{2}\left(1-\alpha \right)\sigma^{2}. \end{array}$$

Thus, we obtain that

$$\mu_{3}=\frac{\left(1-\alpha^{3}\right)\mu^{3}+\left(1+2\alpha^{2}-3\alpha^{3}\right)\mu \sigma^{2}+\alpha^{2}\left(1-\alpha \right)\sigma^{2}}{1-\alpha^{3}}.$$

### Appendix A.2. Proof of Theorem 3

The proof is similar to that of Theorem 4.2 in Cunha et al. (2021) [15]. For estimator $\hat{\alpha }$, we have

$$\begin{array}{cc} \sqrt{T}\left({\hat{\alpha }}_{\mathrm{YW}}-{\hat{\alpha }}_{\mathrm{CLS}}\right)= & \frac{\left(D_{\mathrm{CLS}}-D_{\mathrm{YW}}\right)}{D_{\mathrm{CLS}}D_{\mathrm{YW}}}\cdot \frac{\sum_{t=2}^{T}X_{t}X_{t-1}}{\sqrt{T}}\\ \; & -\frac{\left(D_{\mathrm{CLS}}\sum_{t=2}^{T}\frac{X_{t}}{T}-D_{\mathrm{YW}}\sum_{t=2}^{T}\frac{X_{t}}{T-1}\right)}{D_{\mathrm{CLS}}D_{\mathrm{YW}}}\cdot \frac{\sum_{t=1}^{T}X_{t}}{\sqrt{T}}\\ \; & -\frac{D_{\mathrm{YW}}}{D_{\mathrm{CLS}}D_{\mathrm{YW}}}\cdot \sum_{t=2}^{T}\frac{X_{t}}{T-1}\frac{X_{T}}{\sqrt{T}}+\frac{D_{\mathrm{CLS}}}{D_{\mathrm{CLS}}D_{\mathrm{YW}}}\cdot \bar{X}\frac{\left(X_{T}-\bar{X}\right)}{\sqrt{T}}\\ = & o_{p}\left(1\right)O_{p}\left(1\right)-o_{p}\left(1\right)O_{p}\left(1\right)-O_{p}\left(1\right)O_{p}\left(1\right)o_{p}\left(1\right)+O_{p}\left(1\right)O_{p}\left(1\right)o_{p}\left(1\right)\\ = & o_{p}\left(1\right), \end{array}$$

where $D_{\mathrm{CLS}}=\frac{1}{T}\left[\sum_{t=2}^{T}X_{t-1}^{2}-\frac{1}{T-1}{\left(\sum_{t=2}^{T}X_{t-1}\right)}^{2}\right]$ and $D_{\mathrm{YW}}=\frac{1}{T}\sum_{t=1}^{T}{\left(X_{t}-\bar{X}\right)}^{2}=\frac{1}{T}\sum_{t=1}^{T}X_{t}^{2}-{\bar{X}}^{2}$. For estimator $\hat{\theta }$, we only need to prove $\sqrt{T-1}\left({\hat{\mu }}_{\epsilon ,\mathrm{CLS}}-{\hat{\mu }}_{\epsilon ,\mathrm{YW}}\right)$ is $o_{p}\left(1\right)$.

$$\begin{array}{cc} \; & \sqrt{T-1}\left({\hat{\mu }}_{\epsilon ,\;\mathrm{CLS}\;}-{\hat{\mu }}_{\epsilon ,\mathrm{YW}}\right)\\ = & \sqrt{T-1}\left(\frac{\sum_{t=2}^{T}X_{t}-{\hat{\alpha }}_{\mathrm{CLS}}\sum_{t=2}^{T}X_{t-1}}{T-1}-\bar{X}\left(1-{\hat{\alpha }}_{\mathrm{YW}}\right)\right)\\ = & \frac{1}{\sqrt{T-1}}\left(\sum_{t=2}^{T}X_{t}-{\hat{\alpha }}_{\mathrm{CLS}}\sum_{t=2}^{T}X_{t-1}-T\bar{X}\left(1-{\hat{\alpha }}_{\mathrm{YW}}\right)+\bar{X}\left(1-{\hat{\alpha }}_{\mathrm{YW}}\right)\right)\\ = & \frac{{\hat{\alpha }}_{\mathrm{CLS}}X_{T}-X_{1}}{\sqrt{T-1}}-\sqrt{\frac{T}{T-1}}\cdot \sqrt{T}\left({\hat{\alpha }}_{\mathrm{CLS}}-{\hat{\alpha }}_{\mathrm{YW}}\right)\;\bar{X}+\frac{\bar{X}}{\sqrt{T-1}}\left(1-{\hat{\alpha }}_{\mathrm{YW}}\right)\\ = & o_{p}\left(1\right)-o_{p}\left(1\right)+o_{p}\left(1\right)=o_{p}\left(1\right). \end{array}$$
